# Influence of Aerobic Fitness on White Matter Integrity and Inhibitory Control in Early Adulthood: A 9-Week Exercise Intervention

**DOI:** 10.3390/brainsci11081080

**Published:** 2021-08-18

**Authors:** Hao Zhu, Lina Zhu, Xuan Xiong, Xiaoxiao Dong, Dandan Chen, Jingui Wang, Kelong Cai, Wei Wang, Aiguo Chen

**Affiliations:** 1 College of Physical Education, Yangzhou University, Yangzhou 225127, China; MX120180365@yzu.edu.cn (H.Z.); DX120190066@yzu.edu.cn (X.X.); DX120190065@yzu.edu.cn (X.D.); DX120200078@yzu.edu.cn (D.C.); MX120170362@yzu.edu.cn (J.W.); MX120170353@yzu.edu.cn (K.C.); 2 Institute of Sports, Exercise and Brain, Yangzhou University, Yangzhou 225127, China; zhulina827@mail.bnu.edu.cn; 3 School of Physical Education and Sports Science, Beijing Normal University, Beijing 100875, China; 4 Department of Medical Imaging, The Affiliated Hospital of Yangzhou University, Yangzhou University, Yangzhou 225009, China; 090223@yzu.edu.cn

**Keywords:** diffusion tensor imaging, fractional anisotropy, inhibitory control, aerobic fitness, early adulthood

## Abstract

Previous cross-sectional studies have related aerobic fitness to inhibitory control and white matter (WM) microstructure in young adults, but there is no longitudinal study to confirm whether these relationships exist. We carried out a longitudinal study comparing aerobic fitness, inhibitory control, and WM integrity across time points, before versus after completing an exercise intervention in young adults (18–20 years old) relative to a control group. The exercise group (*n* = 35) participated in a 9-week exercise protocol, while the control group (*n* = 24) did not receive any regular exercise training. Behavioral data and diffusion tensor imaging (DTI) data were collected prior to and following the intervention. After the exercise intervention, aerobic fitness and inhibitory control performance were significantly improved for the exercise group, but not for the control group. Analyses of variance (ANOVA) of the DTI data demonstrated significantly increased fractional anisotropy (FA) in the right corticospinal tract and significantly decreased FA in the left superior fronto-occipital fasciculus in the exercise group after the intervention versus before. The enhanced aerobic fitness induced by exercise was associated with better inhibitory control performance in the incongruent condition and lower FA in the Left superior fronto-occipital fasciculus (SFOF). Regression analysis of a mediation model did not support Left SFOF FA as a mediator of the relationship between improvements in aerobic fitness and inhibitory control. The present data provide new evidence of the relationship between exercise-induced changes in aerobic fitness, WM integrity, and inhibitory control in early adulthood. Longer-duration intervention studies with larger study cohorts are needed to confirm and further explore the findings obtained in this study.

## 1. Introduction

Extensive research involving subjects across various age groups has demonstrated that aerobic fitness can benefit several aspects of cognitive function [1,2,3], including attention [4], inhibitory control [5], memory [6], and executive function [7]. Inhibitory control, which is regarded as a core component of executive function, refers to one’s ability to control his or her attention, behavior, thoughts, and emotions and to overcome his or her strong internal tendencies and external temptations so that he or she is able to initiate and stay engaged in appropriate or necessary tasks [8]. Poorly developed inhibitory control can compromise cognitive, emotional, and social functioning [8]. Thus, a better understanding of the relationship between aerobic fitness and inhibitory control will provide theoretical insights applicable to promoting cognitive development in young people and preventing cognitive decline in the elderly.

To elucidate the relationship between aerobic fitness and inhibitory control, it will be important to clarify how particular brain areas, processes, and inter-regional connections are affected by aerobic fitness and involved in inhibitory control. The right inferior frontal gyrus has emerged as a key region for mediating inhibitory control [9]. The relatively few studies in the literature examining the relationship between fitness and inhibitory control have focused on children or the elderly, and the findings have been ambiguous. A cross-sectional study employing white matter (WM) tractography based on diffusion tensor imaging (DTI) data demonstrated a positive correlation between fitness level and fractional anisotropy (FA) in a cohort of 9- and 10-year-old children [10]. A cross-sectional study involving elderly subjects related fitness to spatial working memory performance, and obtained data suggesting that FA may play a mediating role between aerobic fitness and cognition [11]. Conversely, Herting et al. found no relationship between aerobic fitness and FA in male adolescents [12]. These inconsistent results suggest that the relationship between aerobic fitness, WM integrity, and cognition may vary across different stages of life. 

Although exercise can improve aerobic fitness [13], there is limited information regarding how exercise interventions that enhance aerobic fitness affect WM integrity and cognition. Notably, an intervention study involving 70 participants showed that changes in aerobic fitness induced by 1 year of fitness training were associated with FA changes in prefrontal and temporal cortices as well as with improved short-term memory performance [3]. A recent path analysis study showed that FA could play a mediating role between aerobic fitness and cognition [2]. Although those data were derived from cross-sectional research, the model provides a sound basis from which to examine WM changes associated with the effects of improved fitness on cognition. In general, cross-sectional studies focusing on the relationship between aerobic fitness and WM integrity have lacked cognitive measurements. Moreover, the lack of intervention studies precludes us from making causal inferences about the relationships observed in cross-sectional studies.

Adolescence to early adulthood is a unique developmental period characterized by immature brain processing and vulnerability to psychopathology emergence [14,15]. Although cognitive and WM development changes during this period have slowed relative to earlier developmental phases, ample plasticity remains [16,17]. To the best of our knowledge, the influence of aerobic fitness on WM integrity and behavioral inhibitory control in early adulthood has not been examined in an intervention study. The aim of this study was to examine the influence of aerobic fitness on WM integrity and inhibitory control in young adults, thus addressing this knowledge gap, and to attempt to explore the interaction between the three variables caused by exercise interventions. We hypothesized that after 9-week aerobic exercise intervention, enhanced aerobic fitness would be associated with better inhibitory control performance and alterations in WM integrity based on the supposition that WM integrity may be an important indirect pathway by which aerobic fitness affects inhibitory control. Empirical support for our hypothesis would support the view that aerobic fitness is an important life factor for healthy brain and intellectual development and support the use of DTI-derived FA data for illuminating neural mechanisms underlying exercise effects on brain function.

## 2. Materials and Methods

### 2.1. Participants

A cohort of 84 college freshmen (35 males and 49 females) were recruited from a university in cities of Yangzhou in Jiangsu Province, China. The inclusion criteria were age of 18–20 years old, right-handedness, and normal vision without color blindness. The exclusion criteria were drug abuse, any genetic disease, general intelligence problems, or any medical condition that limits physical activity or that could affect the research results. The study protocol was approved by the ethics and human protection committees of Yangzhou University Affiliated Hospital (2017-YKL045-01). All participants provided written informed consent after receiving a detailed explanation of the experimental procedure. All research procedures were in accordance with the latest version of the Declaration of Helsinki.

### 2.2. Aerobic Fitness Assessment

Peak aerobic oxygen uptake (VO_2_peak) was used as an index of aerobic fitness level [18]. VO_2_peak was assessed while participants exercised on a stress-test stationary bicycle EGT 1000 (ELMED, Zimmer Elektromedizin GmbH in Neu-Ulm, Germany). Before fitness testing, resting heart rate (HR) was determined and then each subject undertook a 3~5-min warm-up to prevent injury. Testing commenced when the subject had returned to his or her resting HR. The testing program started with a 50 W load (a starting load of 50 W was selected to prevent a risk to subjects due to an excessively high initial load) at a cycling speed of 55–60 rotations/min. Maintaining this speed, the load was increased by 50 W every 3 min until the subject reached exhaustion. The subject was considered to have achieved maximum VO_2_ when one of the following criteria was met: (1) no compensatory increase in oxygen intake upon load increase; (2) respiratory quotient exceeded 1.1; (3) HR exceeded 180 beats/min; (4) a speed of 55–60 rotations/min could not be maintained despite repeated encouragement. After the test, the data were recorded. The subjects were instructed to rest for 5 min before being allowed to leave. During this period, a measure of perceived exertion was attained by using the participants’ RPE scale of perceived exertion and they were observed to ensure that they were not having an abnormal reaction.

### 2.3. Exercise Intervention

Participants were randomized into exercise and control groups. Those in the exercise group agreed to take part in exercise training four times a week for 9 weeks. The daily exercise program consisted of a 10-min warm-up, 40 min of moderate-intensity endurance training, and a 10-min cool-down/relaxation period. The exercise program design and implementation were developed and monitored by two trained exercise coaches. We employed a moderate-intensity aerobic load (60–69% of the maximum HR, where maximum HR = 220 − age in years), as defined by the American College of Sports Medicine (2006) [19]. Exercise intensity was assessed with HR monitoring (BHT GOFIT; Beijing, China) the monitors were pre-tested on five subjects before the intervention to ensure the program was appropriate. A return visit was applied to the subjects in the control group. If a subject participated in physical exercise of moderate intensity or above during the intervention period (more than or equal to twice a week), this subject was not included in the final statistical analysis.

### 2.4. Inhibitory Control Testing

Inhibitory control was assessed with a modified Eriksen Flanker task [20]. Briefly, a series of English letters appeared on the screen under two conditions: congruent (FFFFF and LLLLL); and incongruent (LLFLL and FFLFF). Participants were asked to identify the middle letter as quickly as possible by pressing the F key or L key. The two conditions were equally represented and randomly presented. The formal test was composed of two blocks, and each block contained 48 trials, in which the duration of fixation was 500 ms, the duration of letter presentation was 1000 ms, and the stimulation interval was 2000 ms. There were 12 practice sessions before the formal test. A shorter response time (RT) represented better inhibitory control.

### 2.5. Imaging Acquisition

Images were acquired on a 3-T magnetic resonance imaging (MRI) scanner (GE Discovery MR750W) at the Affiliated Hospital of Yangzhou University. None of the subjects had participated in high-intensity physical exercise in the 48 hours prior to the scan. Participants were told to stay relaxed and move as little as possible during the scan. Diffusion images were acquired with an echo planar imaging sequence (acquisition matrix size = 112 × 112, 70 interleaved slices, voxel size = 2 × 2 × 2 mm^3^, field of view 224 × 224 mm^2^, repetition time = 16,500 ms, echo time = 96.2 ms, flip angle = 90°, three B0 images, 30 diffusion weighted images, and a b value of 1000 s/mm^2^).

### 2.6. Image Analysis

PANDA (Pipeline for Analysing braiN Diffusion imAges) (http://www.nitrc.org/projects/panda/), which has automated processing flows, was used to analyze diffusion images [21], including data preprocessing and calculating mean DTI parameter values for whole WM fibers. The preprocessing parameters were: local diffusion homogeneity = 7 voxels; normalizing resolution = 2 mm; and smoothing kernel = 6 mm. The following specific data processes were applied: format conversion, mask generation, image clipping, eddy current and head motion correction, parameter calculation, spatial registration and Gaussian smoothing. Then, using the atlas-based analysis, we normalized FA data in Montreal Neurological Institute space and calculated regional DTI parameters by averaging values within each region of the ICBM DTI-81 atlas [22]. PANDA generates twelve Excel files after completing the automatic processing of data under the folder ‘AllAtlasResults’, which contains the regional average values for diffusion metrics images with a voxel size of 1 × 1 × 1 mm^3^ in the standard space. The values in Excel were then copied to Statistical Package for the Social Sciences (SPSS) for statistics.

### 2.7. Experimental Procedure

A two-factor mixed experiment was carried out with a 2 (groups: exercise and control) × 2 (time: before and after intervention) design. Group was a between-subject factor, while time was a within-subject factor. Data were collected for three periods: pre-intervention test, during the exercise intervention, and post-intervention test. All subjects completed MRI scanning and cognitive testing before the exercise intervention in the pre-test period. One week after completing the pre-intervention test, the exercise group commenced the aforementioned 9-week exercise intervention, while the control group lived normally. After the intervention, all subjects underwent a second MRI scan and cognitive test.

### 2.8. Statistical Analyses

All statistical analysis was implemented in SPSS 22.0 (IBM, Armonk, NY, USA). Mean behavioral test values are reported with standard deviations (SDs). Independent sample *t* tests and χ^2^ tests were used to compare demographic variables across the two groups. Normal distribution test was performed before analysis of covariance. ANOVAs was used to determine whether the two groups differed with respect to change in aerobic fitness before versus after the exercise intervention, with post hoc paired sample t-tests to detect intra-group differences. Repeated-measures analyses of variance (ANOVAs) were performed to analyze effects of the intervention on WM integrity and inhibitory control, with post hoc simple effect analyses. Pearson correlation coefficients (r values) were calculated among changes in aerobic fitness, FA, and inhibitory control. *p* values < 0.05 were considered significant. Causal step regression [23] was used to test whether the influence of aerobic fitness on inhibitory control was mediated by FA. Variables involved in correlation and regression processes were converted into z-scores.

## 3. Results

### 3.1. Participants’ Characteristics

Of the 84 initially enrolled subjects, 59 completed the full study, including 24 freshmen in the control group and 35 freshmen in the exercise group. The demographic data of the exercise group and the control group are presented in Table 1. Due to the loss of data in the control group, there was a significant inter-group difference (*p* < 0.05) in aerobic fitness at baseline. Therefore, we treated aerobic fitness at baseline as a covariate to exclude the influence of baseline differences on the results.

### 3.2. Aerobic Fitness

Treating group as an independent variable, change of aerobic fitness as a dependent variable, and aerobic fitness at baseline as a covariate, an analysis of covariance detected a significant difference in aerobic fitness change between the two groups (F_1,56_ = 37.20, *p* < 0.05, partial η^2^ = 0.571). Paired sample *t*-tests showed that the exercise group showed a significant improvement in aerobic fitness from before (17.96 ± 4.41) to after (30.53 ± 6.45) the exercise intervention (t = −12.03, *p* < 0.05), whereas the control group did not (pre-intervention: 24.07 ± 6.08; post-intervention: 24.49 ± 6.57; t = −0.42, *p* > 0.05). 

### 3.3. Inhibitory Control

Descriptive data for RT and accuracy are presented in Table 2. Two-way Repeated Measures ANOVA, with baseline aerobic fitness as a covariate, revealed a significant group × time interaction in RT in the incongruent condition of the inhibitory control task (F_1,56_ = 25.63, *p* < 0.05, partial η^2^ = 0.314), but not in the congruent condition (F_1,56_ = 0.193, *p* > 0.05, partial η^2^ = 0.003). Simple effect analysis revealed that RT was significantly reduced in the exercise group (*p* < 0.05) and significantly increased in the control group (*p* < 0.05) from the pre-intervention to the post-intervention test. We did not observe a significant group × time interaction in accuracy in the inhibitory control task in either the incongruent (F_1,56_ = 0.121, *p* > 0.05, partial η^2^ = 0.002) or the congruent (F_1,56_ = 0.817, *p* > 0.05, partial η^2^ = 0.014) condition.

### 3.4. WM Structure

**Figure 1:**
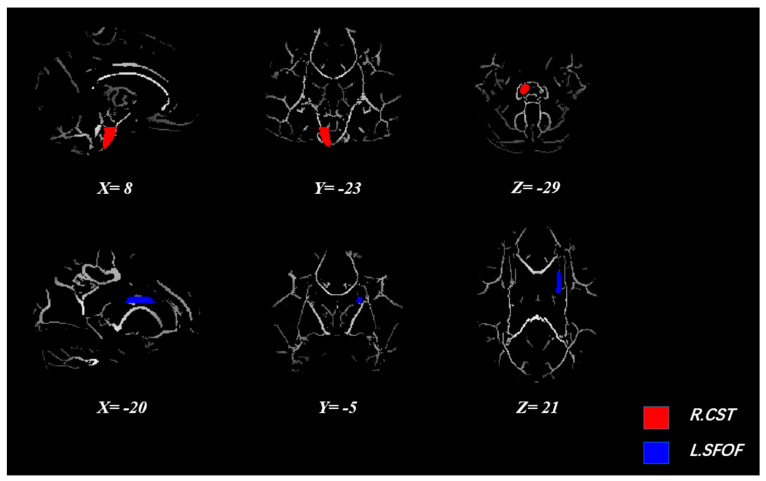
Brain regions with significant interaction effects on FA (*p* < 0.05). Abbreviations: R.CST, right corticospinal tract; L.SFOF, left superior fronto-occipital fasciculus.

Treating aerobic fitness at baseline as a covariate, ANOVAs of FA revealed significant group by time interactions for the right corticospinal tract (Right CST) (F_1,56_ = 4.033, *p* < 0.05, partial η^2^ = 0.067) and left superior fronto-occipital fasciculus (Left SFOF) (F_1,56_ = 5.682, *p* < 0.05, partial η^2^ = 0.092) (Figure 1). Simple effect analysis indicated that the FA of the right corticospinal tract presented an upward trend in the exercise group, but a downward trend in the control group. Meanwhile, FA of the Left SFOF showed a downward trend and an upward trend in the exercise group and control group, respectively. The specific FA values of the brain regions with significant interactions are listed in Table 3.

### 3.5. Changes in Aerobic Fitness, FA, and Inhibitory Control

Aerobic fitness correlated inversely with FA of the L.SFOF (r = −0.26, 95% CI (−0.473, 0.032), *p* < 0.05), with increases in aerobic fitness being accompanied by L.SFOF FA decreases, but did not correlate significantly with FA of the right corticospinal tract (r = 0.06, *p* > 0.05). Additionally, aerobic fitness correlated inversely with RT in the inhibitory control task (r = −0.45, 95% CI (−0.657, −0.213), *p* < 0.05), indicating that a higher aerobic fitness level was predictive of better inhibitory control performance. Regression analysis of the mediation model wherein aerobic fitness change and FA change of left superior fronto-occipital fasciculus were treated as independent variables, while inhibitory control change was treated as a dependent variable, yielded a non-significant (*p* > 0.05) regression coefficient (−0.024), thereby invalidating the FA mediation model.

## 4. Discussion

The present study demonstrated that a 9-week aerobic exercise intervention, which was confirmed to improve aerobic fitness, could induce changes in WM integrity and inhibitory control in young adults. Specifically, the exercise group exhibited improved inhibitory control together with increased FA in right corticospinal tract and decreased FA in left superior fronto-occipital fasciculus. The exercise-induced changes in aerobic fitness correlated with observed effects on inhibitory control and FA of the Left SFOF. However, regression analysis indicated that the altered FA of the Left SFOF, examined as a potential mediator, could not account for the relationship between aerobic fitness and inhibitory control.

### 4.1. Behavior

Three prior meta-analyses focusing on different age groups [24,25,26] showed that long-term participation in aerobic exercise improved aerobic fitness in both healthy participants and patients with mental illness [27] and cancer [28]. Our findings reinforce the notion that exercise interventions improve aerobic fitness, specifically during early adulthood.

The view that exercise promotes inhibitory control has been supported by behavioral, physiological, and imaging studies [29,30,31]. The exercise paradigm used in the present study yielded RT improvement selectively in the incongruent condition of the Eriksen Flanker task, without a concomitant significant improvement in accuracy. Prior studies in children and in the elderly showed RT improvements in both incongruent and congruent conditions. The more limited improvement in our study could be related to the ages of the subjects. That is, in early adulthood, which is not long after puberty, brain morphology and core cognitive abilities are more firmly established than in children, whereas elderly participants are more likely to have acquired some mild cognitive deficits that may benefit from exercise [32]. Furthermore, only the relatively more difficult of the conditions in the Eriksen Flanker task, which is a relatively simple task, may have been sufficiently sensitive to reflect a mild improvement in inhibitory control. In general, our behavioral data confirm that exercise can improve aerobic fitness and inhibitory control in early adulthood.

### 4.2. WM Integrity

WM, which is composed of glial cells and myelinated neurons, matures mostly before early adulthood, and myelin formation contributes to efficient neurotransmission throughout the brain, which in turn improves cognitive function [12,33]. The most commonly examined index of WM integrity is FA, with larger FA values representing greater diffusion of water molecules in the axial direction than that in the radial direction within analyzed WM tracts. Overweight children who participated in an exercise intervention were found to have a significant increase in bilateral uncinate fasciculus FA after completing an 8-month exercise program [34]. A recent study showed that a 12-week badminton intervention increased FA in the posterior limb of the internal capsule and upper corona radiate in participants in their mid-twenties [35]. The present study showed distinct sites of WM changes relative to the sites of WM changes observed in these prior studies. Notwithstanding, the increased FA of the right corticospinal tract observed in our study is consistent with the findings of a prior cross-sectional study [12]. However, our finding of reduced FA of the left superior fronto-occipital fasciculus after the exercise intervention was unexpected. It might be related to the age of our subjects (18 to 20 years old). FA in tracts of the frontal lobe peaks in late adolescence and 50% of corticospinal tract voxels reach their FA peak at about 20 years old, followed by downward trends [36]. Thus, because the FA of the left superior fronto-occipital fasciculus in our subjects would be at or near peak developmental values, it may difficult to augment with an intervention. Alternatively, a meta-analysis suggested that open-skill exercises may be more effective than closed-skill exercises for mobilizing cognitive load and promoting inhibitory control improvement [37]. Badminton is an open-skill exercise wherein one needs to respond quickly to a dynamic and unpredictable environment. Here, we employed a closed-skill exercise paradigm that was relatively consistent and well controlled. Hence, WM integrity may be differentially influenced under different exercise intervention treatments. The presently employed 9-week exercise intervention reshaped the WM integrity in young adults, an age group for which data were lacking. Longer-term studies are needed to clarify how exercise and fitness improvement affect WM.

### 4.3. The Relationship between Improved Aerobic Fitness and Inhibitory Control Cannot Be Explained by FA Alterations

Pair-wise correlation analysis demonstrated a significant correlation between increased aerobic fitness and decreased RT in the Eriksen Flanker task. This finding fits with prior research showing that individuals with high aerobic fitness perform better than less fit individuals on various cognitive tasks. With respect to mechanism, authors of prior studies have suggested that this relationship may be related to effects on brain functional connectivity [38], prefrontal cortex thickness [39], and event-related potentials [5]. The present work adds evidence of WM integrity changes to the potential mechanisms that may contribute to aerobic fitness effects on cognitive performance.

Both FA of the right corticospinal tract and FA of the left superior fronto-occipital fasciculus were found to have significant interaction effects in this study, with FA of the left superior fronto-occipital fasciculus having a significant negative correlation with aerobic fitness, while FA of the right corticospinal tract had only a non-significant trend toward a positive correlation with aerobic fitness. By contrast, previous cross-sectional studies have shown significant positive correlations between aerobic fitness and FA in adolescents with a mean age of 14.3 years ([40] and in (older) young adults with a mean age of 28.8 years [2]). The present study provides data for an intermediate group (mean age, 18.6 years), thus supplementing the literature. The relationship between the changes in aerobic fitness and changes in FA induced by an exercise intervention has not been examined in very early adulthood previously. Meanwhile, our novel finding of a negative correlation between aerobic fitness and FA of the left superior fronto-occipital fasciculus might be explained by a reduction of FA in association with gaining fluency with an automated movement [41,42,43,44]. Furthermore, we found no correlation between changes in FA and RT reduction in the Eriksen Flanker task. This negative finding may be due to our relatively small sample size or short intervention duration. Recent studies have been inconsistent, with one study showing a positive correlation between FA and inhibitory control [45], and another showing that higher FA was associated with worse inhibitory control performance [46]. Future studies with more rigorous experimental designs are needed to clarify whether there is specificity regarding exercise interventions that lead to associations between FA and aerobic fitness.

Our supposition that FA of the left superior fronto-occipital fasciculus may play a mediating role in the influence of aerobic fitness on inhibitory control was not confirmed, which may be due to the short duration of intervention or the specific characteristics of the structural development phase of the brain in early adulthood. Early adulthood is a highly plastic cognitive developmental period when people transition from secondary school to social and economic independence, and thus is of particular interest for educational decision-makers and researchers. Notably, WM developmental properties during this phase of life are distinct from those during childhood, when there is more rapid WM development, and also distinct from those during old age, when there are declines in WM integrity [47,48].

### 4.4. Strengths and Limitations

A major advantage of our study is that an exercise intervention was employed as a breakthrough point to explore the relationship between increased aerobic fitness, changes in WM integrity, and inhibitory control improvement in early adulthood, extending the results of previous cross-sectional studies. The conclusions of this study should be considered in light of several limitations. First, aerobic fitness at baseline differed between the exercise and control groups due to loss of subjects. Although aerobic fitness at baseline was treated as a statistical covariate, it still could have impacted the accuracy of the results. In addition, the loss of subjects leads to the reduction of sample size, which can be avoided as far as possible in future studies to improve the reliability of the study. Second, there are additional indicators of WM integrity that were not analyzed in our study, including mean diffusivity, radial diffusivity, and axial diffusivity. Further exploration of these indicators could further clarify the relationships among aerobic fitness, WM integrity, and cognition. Third, prior studies involving children [49] and elderly participants [3] adopted longer term interventions (8 months and 12 months, respectively) than that used in this study. It is possible that the 9-week intervention examined here was not sufficiently long in duration to reveal a significant positive relationship between aerobic fitness and FA of the right corticospinal tract. Therefore, the effects of higher exercise doses (duration, intensity, and frequency) should be examined in the future. Finally, we only compared data across two time points (pre-intervention and post-intervention). Because exercise-induced FA changes may be nonlinear, future research should collect data at multiple time points during the intervention process.

## 5. Conclusions

The present results showed that a 9-week exercise intervention that improves aerobic fitness resulted in an improvement in inhibitory control performance and changes in WM integrity in very young adults. Enhanced aerobic fitness correlated positively with an inhibitory control performance parameter and correlated negatively with FA of the left superior fronto-occipital fasciculus. However, a mediator role of altered FA in the relationship between improved aerobic fitness and improved inhibitory control could not be demonstrated. This work provides a reference for future research exploring the impact of aerobic fitness on cognition from the perspective of WM integrity.

## Figures and Tables

**Table 1:** Participant demographics (M ± SD).

Variable	Control Group	Exercise Group	*p*
*N*	24	35	
male/female	9/15	15/20	0.68
age	18.5 ± 0.78	18.66 ± 0.48	0.39
BMI	22.46 ± 4.28	20.8 ± 2.22	0.09
aerobic fitness at baseline	24.07 ± 6.08	17.96 ± 4.41	0.00
HR during treatment		136.64 ± 7.77	

**Table 2:** Mean RTs and accuracy rates (M ± SD) in the Eriksen Flanker task by group.

Variable	Control Group (*N* = 24)		Exercise Group (*N* = 35)	
	Pre-Intervention	Post-Intervention	Pre-Intervention	Post-Intervention
**RT, ms**				
Congruent	486.24 ± 50.11	451.78 ± 61.34	501.39 ± 99.28	473.78 ± 48.40
Incongruent *	520.85 ± 41.96	560.96 ± 63.15	547.49 ± 48.36	507.09 ± 38.53
**Accuracy, %**				
Congruent	95.57 ± 4.04	95.23 ± 5.05	95.77 ± 3.70	95.99 ± 3.15
Incongruent	93.84 ± 4.49	95.14 ± 5.20	94.32 ± 3.71	95.84 ± 3.23

* There is a significant group × time interaction (*p* < 0.05) under the incongruent condition.

**Table 3:** FA values of the brain regions with interaction (M ± SD).

Brain Region	Control Group (*N* = 24)		Exercise Group (*N* = 35)	
	Pre-Intervention	Post-Intervention	Pre-Intervention	Post-Intervention
Right CST	0.561 ± 3.20 × 10^−2^	0.551 ± 3.07 × 10^−2^	0.557 ± 3.69 × 10^−2^	0.562 ± 3.65 × 10^−2^
Left SFOF	0.460 ± 3.11 × 10^−2^	0.470 ± 3.19 × 10^−2^	0.472 ± 3.20 × 10^−2^	0.465 ± 2.47 × 10^−2^

Note: Right CST, right corticospinal tract; Left SFOF, left superior fronto-occipital fasciculus.
